# Estimation of Land Surface Temperature in an Agricultural Region of Bangladesh from Landsat 8: Intercomparison of Four Algorithms

**DOI:** 10.3390/s20061778

**Published:** 2020-03-23

**Authors:** Md Qutub Uddin Sajib, Tao Wang

**Affiliations:** School of Environmental Studies, China University of Geosciences, Wuhan 430078, China; qsajib71@gmail.com

**Keywords:** remote sensing, land surface temperature, emissivity, NDVI, intercomparison

## Abstract

The presence of two thermal bands in Landsat 8 brings the opportunity to use either one or both of these bands to retrieve Land Surface Temperature (LST). In order to compare the performances of existing algorithms, we used four methods to retrieve LST from Landsat 8 and made an intercomparison among them. Apart from the direct use of the Radiative Transfer Equation (RTE), Single-Channel Algorithm and two Split-Window Algorithms were used taking an agricultural region in Bangladesh as the study area. The LSTs retrieved in the four methods were validated in two ways: first, an indirect validation against reference LST, which was obtained in the Atmospheric and Topographic CORection (ATCOR) software module; second, cross-validation with Terra MODerate Resolution Imaging Spectroradiometer (MODIS) daily LSTs that were obtained from the **App**lication for **E**xtracting and **E**xploring **A**nalysis **R**eady **S**amples (AρρEEARS) online tool. Due to the absence of LST-monitoring radiosounding instruments surrounding the study area, in situ LSTs were not available; hence, validation of satellite retrieved LSTs against in situ LSTs was not performed. The atmospheric parameters necessary for the RTE-based method, as well as for other methods, were calculated from the National Centers for Environmental Prediction (NCEP) database using an online atmospheric correction calculator with MODerate resolution atmospheric TRANsmission (MODTRAN) codes. Root-mean-squared-error (RMSE) against reference LST, as well as mean bias error against both reference and MODIS daily LSTs, was used to interpret the relative accuracy of LST results. All four methods were found to result in acceptable LST products, leaving atmospheric water vapor content (*w*) as the important determinant for the precision result. Considering a set of several Landsat 8 images of different dates, Jiménez-Muñoz et al.’s (2014) Split-Window algorithm was found to result in the lowest mean RMSE of 1.19 ${}^{{}^{\circ}}C$. Du et al.’s (2015) Split-Window algorithm resulted in mean RMSE of 1.50 ${}^{{}^{\circ}}C$. The RTE-based direct method and the Single-Channel algorithm provided the mean RMSE of 2.47 ${}^{{}^{\circ}}C$ and 4.11 ${}^{{}^{\circ}}C$, respectively. For Du et al.’s algorithm, the *w* range of 0.0 to 6.3 g cm^−2^ was considered, whereas for the other three methods, *w* values as retrieved from the NCEP database were considered for corresponding images. Land surface emissivity was retrieved through the Normalized Difference Vegetation Index (NDVI)-threshold method. This intercomparison study provides an LST retrieval methodology for Landsat 8 that involves four algorithms. It proves that (i) better LST results can be obtained using both thermal bands of Landsat 8; (ii) the NCEP database can be used to determine atmospheric parameters using the online calculator; (iii) MODIS daily LSTs from AρρEEARS can be used efficiently in cross-validation and intercomparison of Landsat 8 LST algorithms; and (iv) when in situ LST data are not available, the ATCOR-derived LSTs can be used for indirect verification and intercomparison of Landsat 8 LST algorithms.

## 1. Introduction

Estimation of Land Surface Temperature (LST) and the study of its changes over time is an important topic of research because, these days, global climate is changing fast. Therefore, retrieval of LST with new technologies has become an interesting field to explore in order to better understand the environment all over the world. With the recent advancement in remote sensing earth observation systems, studying LST, as well as land use and land cover (LULC), has become much easier than it was before. Today, many sources of satellite images are available containing optical, as well as thermal, information of earth surfaces.

LST is the thermodynamic skin temperature of land surfaces which can be studied by measuring the infrared radiation coming from the surface [1]. With LST information, urban heat island can be monitored [2,3] and forest fire can be detected [4]. LST information can be useful to estimate the soil moisture [5,6,7]; hence, studies related to many hydrological processes can be explored from LST [8]. It can also help in different climate studies and weather forecast [8,9,10]. Changes in LST over time can be related with changes in LULC types [11]. LST is related with all sorts of processes that control the energy and water fluxes over the interfaces between the Earth’s surface and the atmosphere [12,13]. All these applications make the study of LST a crucial parameter to better understand the regional, as well as the global, environment and its changes over time.

There are different algorithms proposed for the retrieval of LST using different sources of Remote Sensing (RS) data [14,15,16,17,18,19,20]. Among those sources, Landsat has the biggest archive of free images and are of great interest among researches for LST study. Studies with Landsat data include LST retrieval with Landsat 5 TM (Thematic Mapper) data over an agricultural region of Spain by Sobrino et al. [21]; LST with Landsat 7 ETM+ (Enhanced Thematic Mapper Plus) data over Maraqeh County of Iran [22]; and, a work by Fu and Weng [23] for consistent and daily LST monitoring using Landsat images from 1984 to 2011 over the Beijing city of China. Mallick et al. [24] used Landsat 7 ETM+ data to perform LULC and LST study over a heterogeneous urban area of India. Sahana et al. [25] studied LULC change and its impact on LST using Landsat 5 TM data and Landsat 8 thermal infrared (TIR) data over the Sundarban biosphere reserve in India.

Study of LST with RS data over different areas in Bangladesh is rather limited compared to other countries of the world. Bangladesh covers an area of 147,570 km${}^{2}$ [26] with a huge population. Geographically it extends from 20°34′ N to 26°38′ N latitude and from 88°1′ E to 92°41′ E longitude [26]. It is one of the most densely populated countries in the world [27]. Most of its population live in its capital city. An agricultural region close to the capital city was selected as the study area for this work (see Section 2.2).

Speaking of the limited LST research works in Bangladesh, those found in literature include the capital city and some of its surrounding areas [28,29,30], and at least one work covering the whole country [31]. Changes in LST and land cover over time, as well as future LST simulation, was studied by Ahmed et al. [28] in the capital city of Bangladesh using Landsat 4, 5 TM, and Landsat 7 ETM+ data. Reja [29] retrieved LST using Landsat 4 TM and Landsat 7 ETM+ data. Sultana et al. [31] used NOAA-16 (National Oceanic and Atmospheric Administration - 16) and NOAA-17 data to estimate the minimum and maximum LSTs for six different seasons of whole country. Roni [30] studied the relation between LST and Normalized Difference Vegetation Index (NDVI) using Landsat TM data. Ara et al. [32] used Landsat 8, along with Landsat TM and ETM+ data, to study the effects of land use intensity on LST in Chittagong city corporation area. Study of LST with Landsat 8 images in the capital city or its surrounding areas in Bangladesh is not known as of the time of writing this paper.

Retrieval of LST with the precision result depends on data type, environmental conditions, and the particular algorithm used for the calculation. LST retrieval algorithms may depend on the presence of one or multiple thermal bands in the source RS data. Thermal channel in electromagnetic spectrum covers the region of 10 to 12 μm. Previous Landsat missions came with only one thermal channel but Landsat 8 has two. Sensors with more than one thermal band allow the user to extract information from both of these bands with the possibility of better LST retrieval.

Geostationary satellites/platforms, such as SEVIRI (Spinning Enhanced Visible and Infrared Imager), can be used to retrieve surface emissivity and temperature simultaneously by using Kalman filter strategy [33]. The retrieval process is physical-based and can be applied for both land- and sea-surface temperatures with very good results compared with LST from non-geostationary satellite observations [34]. The same Kalman filter methodology can be found to produce a very good result in different land cover types, including arid, cultivated, and vegetated, as well as urban, areas and sea water [35].

In this study, we used four algorithms—a Single-Channel Algorithm [36,37], two Split-Window algorithms [37,38,39,40], and a direct method based on the Radiative Transfer Equation (RTE) [41,42]—to retrieve LST from Landsat 8 data. All these algorithms were used for LST retrieval on a study area covered mostly with vegetative land surfaces. Located in Bangladesh with subtropical climatic conditions, the selected area during the period of study (February 2018) experiences pleasantly sunny winter with minimal to no precipitation and mostly clear to partly cloudy sky conditions. All four algorithms for Landsat 8 in this study area were found providing with acceptable LST results compared with reference LSTs and MODerate Resolution Imaging Spectroradiometer (MODIS) daily LSTs, while the Split-Window algorithms performed better than the other two (see Section 4). Based on the LST results from the four methods, an intercomparison among them was made. A cross-validation study of LSTs obtained from Landsat 8 images was performed against MODIS daily LST data. At  1 km spatial resolution, the MODIS images produce global daily LST data [43]; hence, it can be used for cross-validation of Landsat LST products. Available online at https://lpdaacsvc.cr.usgs.gov/appeears/, the **App**lication for **E**xtracting and **E**xploring **A**nalysis **R**eady **S**amples (AρρEEARS) can be used to extract daily or 8-day composite MODIS LSTs [44,45].

This paper is organized as follows: Section 2 describes materials and methods, including the details about remote sensing data used in this study. A unified methodology flowchart involving different steps in LST retrieval with four algorithms is also presented in this section. Section 3 provides the theoretical aspects of LST study from Landsat 8 with brief description of four algorithms. It also describes the estimation of normalized difference vegetation index, proportion of vegetation cover, land surface emissivity, top-of-atmosphere (ToA) brightness temperature, and the processing of input data for LST algorithms. Section 4 presents the results of LSTs retrieved with four algorithms, including their validation results against reference LST, as well as the intercomparison study, among them. Results from the cross-validation study of Landsat 8 LSTs against MODIS daily LSTs are also presented in this section. Section 5 summarizes the findings and concluding remarks of this work.

Mathematical notations of parameters involved in LST determination are found in literature with not-so-uniform expressions. In this paper, $T_{\mathrm{ToA},i}$ denotes the top-of-atmosphere (ToA, or at-sensor) brightness temperature for channel *i*; $L_{\mathrm{ToA},i}$ is the ToA spectral radiance for channel *i*; $B_{i}\left(T_{i}\right)$ is the ToA thermal radiance; $\rho_{\lambda ,\mathrm{LS}}$ is the reflectance from the surface; LSE${}_{i}$ is the Land Surface Emissivity for band *i*; and $P_{v}$ is the proportion of vegetation. Because of the stray light effect observed in band 11 images of Landsat 8 [46,47], we used RTE-based direct method and Single-Channel method for band 10 only. In Split-Window algorithms, however, both thermal bands were used.

## 2. Materials and Methods

Landsat 8 images were used as the primary sources of data to retrieve the LST products. Two Split-Window algorithms, a Single-Channel Algorithm, and an RTE-based direct method were used. The Landsat 8 satellite-retrieved LST products obtained in four methods were validated against reference LSTs and MODIS daily LSTs. The idea is to perform an intercomparison, and study the relative performances of four existing LST algorithms from Landsat 8. The reference LST was retrieved with the Atmospheric and Topographic CORection (ATCOR) module. The MODIS daily LST products were extracted using the AρρEEARS online tool. A study area in an agricultural region of Bangladesh was selected for this research.

### 2.1. Dataset

Landsat 8, the primary source of data in this work, travels on the descending (daytime) node from north to south and crosses the equator at 10:11 a.m. ±15 min mean local time. For our study area, primarily one Landsat 8 image was downloaded from the USGS earth explorer website. The image was taken on 21 February 2018—the actual date on which the authors conducted a field work in the study area—with Operational Land Imager (OLI) and Thermal Infrared (TIR) scanners onboard Landsat 8, and has Path 137 and Row 44. The TIR bands were used to retrieve LSTs, along with the red (band 4) and infrared (band 5) channels for estimating NDVI. The shapefiles necessary for our study were downloaded from the Database of Global Administrative Areas (GADM—https://gadm.org/).

In order to test the LST algorithms on other Landsat 8 images, a set of four Landsat 8 scenes of different dates were downloaded. Two of them were images taken before 21 February 2018, while the other two images were taken after 21 February. We did not use the image of 5 February 2018 because this image was found covered with 77.4% cloud. The amount of cloud cover in percentage is available in the Landsat 8 image metadata file as CLOUD_COVER. The approach used for the cloud classification in Landsat 8 images involve multiple algorithms. It is collectively known as the Cloud Cover Assessment (CCA) and includes the Automated Cloud Cover (ACCA), See-5 CCA, Cirrus CCA, AT-ACCA, etc., algorithms [48] to classify clouds. The CCA analysis results are then merged into the final L1 quality band and the cloud cover amount is made available in the image metadata file. Further details regarding the cloud detection algorithms of Landsat 8 can be found in Reference [48].

To perform cross-validation of Landsat 8 LST products with MODIS daily LST data, we downloaded Terra MODIS LST products using the AρρEEARS online application. We extracted the daily MODIS LSTs of different dates by uploading the shapefiles of our study area in the online module. Details of all Landsat 8 and MODIS images used in this study are presented in Table 1.

### 2.2. Study Area

The study area selected for the verification and intercomparison of four LST algorithms is Chandina sub-district under Cumilla district in Bangladesh. Cumilla district is adjacent to the capital city of Bangladesh. Located in South Asia, Bangladesh is virtually surrounded by India and the Bay of Bengal to the south [49]. It is a low-lying country with huge count of rivers.

**Figure 1:**
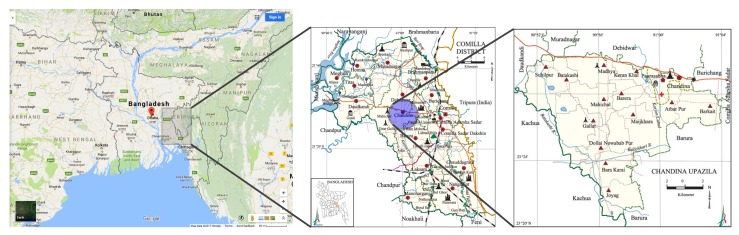
Chandina sub-district of Cumilla district in Bangladesh (from left to right, not to scale). Compiled from References [50,51,52].

Cumilla district is situated in the south eastern part of Bangladesh [50]. It has an area of 3085.17 km${}^{2}$, and located in between 23°2′ N to 24°47′ N latitudes and in between 92°39′ E to 91°22′ E longitudes [51]. There are 17 *Upazilas* (sub-districts) in this district, among which Chandina is one. Figure 1 shows the location of this sub-district as our study area.

Chandina sub-district is an area of about 201 km${}^{2}$, with mostly vegetative land surfaces. A visit to the study area was arranged as part of this research, and it was found that different types of vegetables are grown in this area.

The study area observation was conducted in two consecutive days, 21 and 22 February in 2018, under clear sky conditions, starting the visit in the morning at around 8:30 local time till 15:30 in the afternoon. There were no radiosounding instruments available surrounding the study area; hence, in situ LST data were not monitored. It was found that the local experts usually monitor the LST with soil thermometers in these areas, which may not be a good measurement of in situ LSTs compared to radiometer-retrieved in situ LSTs. Therefore, the intercomparison study of four LST algorithms from Landsat 8 was carried out against ATCOR-derived reference LSTs and AρρEEARS-derived MODIS daily LSTs.

### 2.3. Research Methodology

**Figure 2:**
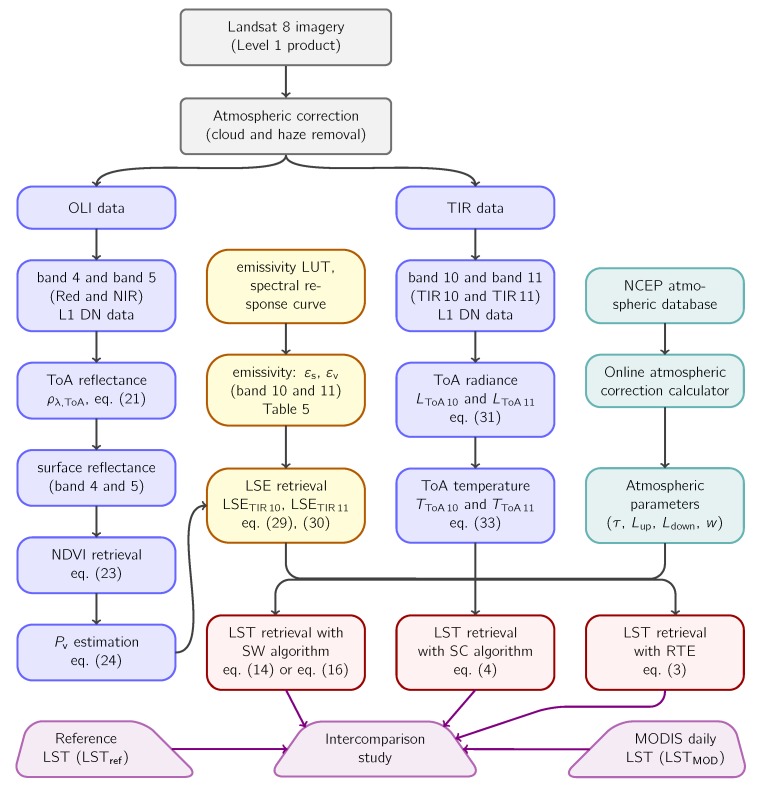
Algorithm flowchart for Land Surface Temperature (LST) retrieval with four methods and their intercomparison study from Landsat 8 thermal infrared (TIR) data. OLI = Operational Land Imager; LUT = Look Up Table; DN = Digital Number; NDVI = Normalized Difference Vegetation Index; SW = Split-Window; NCEP = National Centers for Environmental Prediction; ToA = Top-of-Atmosphere; NIR = Near-Infrared; LSE = Land Surface Emissivity; SC = Single-Channel; TIR = Thermal Infrared.

The methodology to perform the intercomparison of different LST algorithms requires the estimation of LSTs from Landsat 8 images using each of these algorithms. A flowchart representing different steps in LST estimation with four algorithms is shown in Figure 2. As shown in this flowchart, processing of the remote sensing data starts with the level-1 product of Landsat 8 images. Then, the first step is to remove the cloud and haze from level-1 DN values, which is a part of the atmospheric correction. In this study, we used the ATCOR module for atmospheric correction, which uses MODerate resolution atmospheric TRANsmission (MODTRAN) codes to classify and remove cloud and haze from a Landsat 8 scene.

Once the remote sensing images are atmospherically corrected, both optical (OLI) and thermal (TIR) bands are necessary to determine the LST. Using optical bands, band 4 (red) and band 5 (infrared) in particular, NDVI is estimated. From the NDVI, first the proportion of vegetation ($P_{v}$), then land surface emissivity (LSE) is determined.

Using both thermal bands of Landsat 8 data, top-of-atmosphere radiance ($L_{\mathrm{ToA}}$) is first determined. Then, top-of-atmosphere temperature ($T_{\mathrm{ToA}}$) is estimated. After that, using appropriate LST algorithm with its mathematical formula and its coefficient values (see Section 3), LST can be retrieved using emissivity and $T_{\mathrm{ToA}}$ as shown in the flowchart.

Once LSTs with different algorithms are estimated, they can be validated against in situ LSTs or reference LSTs. In this study, the algorithm-retrieved LSTs from Landsat 8 were validated comparing them with reference LSTs retrieved using the ATCOR module. Validation of algorithm-retrieved LST against ATCOR-retrieved LST can be called as indirect verification [53]. The indirect verification was chosen because the in situ LST data were not available due to the absence of radiosounding instruments in the study area. In addition to the indirect verification, cross-validation of Landsat 8 LSTs obtained from different algorithms was performed comparing them with MODIS daily LSTs. Intercomparison study of four Landsat 8 LST algorithms was performed against reference LSTs and MODIS daily LSTs.

## 3. Four Methods to Retrieve Land Surface Temperature from Landsat 8

The thermal channels of Landsat 8 images are band 10 and band 11, also known as TIR 1 and TIR 2 channels, respectively. First of these two has thermal window of 10.60 to 11.19 μm, and the latter 11.50 to 12.51 μm [48]. Since objects or land surfaces transmit radiation in different amounts depending on the wavelength of the channel window, reflected radiation recorded from two thermal bands contain different information about the land surface.

Retrieval methods of LST from thermal bands are based on the Radiative Transfer Equation (RTE). This equation can be used directly to estimate LST; or Single-Channel, Split-Window etc. methods can be used. The RTE-based direct method requires the information of atmospheric parameters for the study area. On the other hand, Single-Channel or Split-Window methods do not require the direct input of those parameters but use some coefficient values obtained through simulation calculations.

While LST can be estimated using one thermal band, the calculation of differential absorption in multiple TIR bands minimizes the atmospheric effects and has the potential to provide better results compared to the use of one thermal band [54]. This idea of using more than one thermal bands is called Split-Window technique [10,14]. Split-Window algorithms for Landsat 8 thermal bands were proposed by many researchers including Rozenstein et al. [55], Jiménez-Muñoz et al. [37], Yu et al. [41], and Du et al. [40]. In this study, we used Split-Window algorithms developed by Jiménez-Muñoz et al. [37] and by Du et al. [40] because: (i) these two algorithms were found providing good performances in different *w* ranges [40], and (ii) first of these two requires direct input of *w* value, whereas the second comes with algorithm coefficients for several *w* sub-ranges. Therefore, it would be interesting to see how these two algorithms perform by making use of *w* in different ways.

When a Single-Channel or Split-Window algorithm is developed, using the MODerate resolution atmospheric TRANsmission (MODTRAN) or other radiative transfer codes, algorithm coefficients are estimated and made available to users so that they can be input directly in the mathematical expression of the algorithm to study LST for different areas of the world. Among various atmospheric profile databases that can be used in MODTRAN codes, examples include the Thermodynamic Initial Guess Retrieval (TIGR) profile constructed by the Laboratoire de Météorologie Dynamique [40], the Global Atmospheric Profiles from Reanalysis Information (GAPRI) database [37,56], the SAFREE database and the Cloudless Land Atmosphere Radiosounding (CLAR) database [56], the National Centers for Environmental Prediction (NCEP) database [57], etc.

The MODTRAN radiative transfer codes take atmospheric parameters and surface parameters as inputs. Spectral parameters needed for the MODTRAN codes are obtained from the spectral response functions of given TIR sensors [37,40,58]. Surface parameters for the MODTRAN codes may include emissivity [57], viewing geometry, etc. The atmospheric parameters retrieved from these codes include the transmittance (τ), up-welling path radiance ($L_{\mathrm{up}}$), and down-welling path radiance ($L_{\mathrm{down}}$) for the given thermal channel. In the following, we provide short description of four LST retrieval methods for Landsat 8 that were used for intercomparison in this study.

### 3.1. Radiative Transfer Equation and Atmospheric Parameters to Retrieve Land Surface Temperature

The formula to retrieve land surface temperature using Radiative Transfer Equation (RTE) can be expressed as [41]:

(1) $$\mathrm{LST}=\frac{c_{2}}{\lambda_{i}\;\cdot \;\ln\left(\frac{c_{1}\;\cdot \;\tau_{i}\varepsilon_{i}}{\lambda_{i}^{5}\;\cdot \;\left(B_{i}\left(T_{i}\right)-L_{\mathrm{up}}-\tau_{i}\left(1-\varepsilon_{i}\right)\;\cdot \;L_{\mathrm{down}}\right)}+1\right)},$$

where $\lambda_{i}$ is the effective wavelength of band *i*; $B_{i}\left(T_{i}\right)$ is the ToA thermal radiance, $\tau_{i}$ is the band average atmospheric transmittance, and $\varepsilon_{i}$ is the emissivity of the same band; $L_{\mathrm{up}}$ and $L_{\mathrm{down}}$ are the upwelling and downwelling radiance in the atmosphere obtained in band *i*; $c_{1}$ and $c_{2}$ are Planck’s first and second radiation constants, respectively, with $c_{1}=1.19104\times 10^{8}\;W\;\mu m^{4}m^{-2}{\mathrm{sr}}^{-1}$ and $c_{2}=1.43877\times 10^{4}\;\mu m\;K$. To retrieve LST with RTE using Equation (1) three atmospheric parameters are needed: $\tau_{i}$, $L_{\mathrm{up}}$, and $L_{\mathrm{down}}$; besides these parameters, land surface emissivity ($\varepsilon_{i}$) is necessary.

**Figure 3:**
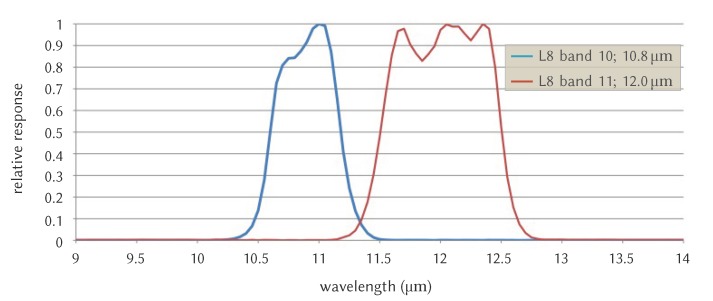
Effective wavelengths of Landsat 8 TIRS bands; 10.8 μm for TIR band 10, and 12 μm for TIR band 11. Source: Reference [59] (relabeled by the authors).

The $\lambda_{i}$ in Equation (1) (or $\lambda_{\mathrm{eff}}$) for a given band can be estimated as [41,42]:

(2) $$\lambda_{\mathrm{eff}}=\frac{\int_{\lambda_{1}}^{\lambda_{2}}\lambda \;f\left(\lambda \right)\;d\lambda }{\int_{\lambda_{1}}^{\lambda_{2}}f\left(\lambda \right)\;d\lambda },$$

where f(λ) is calculated as the function of the spectral responsivity of thermal bands; $\lambda_{1}$ and $\lambda_{2}$ are the lower and upper limit of f(λ). As shown in Figure 3, the $\lambda_{\mathrm{eff}}$ for Landsat 8 band 10 is 10.8 μm and for band 11 is 12 μm.

For Landsat 8 TIR band 10, Equation (1) can be rewritten as

(3) $$\mathrm{LST}=\frac{c_{2}}{\lambda_{\mathrm{eff},\mathrm{TIR}10}\;\cdot \;\ln\left(\frac{c_{1}\;\cdot \;\tau_{\mathrm{TIR}10}\;\cdot \;{\mathrm{LSE}}_{\mathrm{TIR}10}}{\lambda_{\mathrm{eff}}^{5}\;\cdot \;\left(L_{\mathrm{ToA}\;10}-L_{\mathrm{up}}-\tau_{\mathrm{TIR}10}\left(1-{\mathrm{LSE}}_{\mathrm{TIR}10}\right)\;\cdot \;L_{\mathrm{down}}\right)}+1\right)}.$$

The atmospheric parameters needed to retrieve LST using Equation (3) can be estimated with local radiosounding instruments (if available) or from global atmospheric profiles using simulation codes.

The online atmospheric correction calculator used in this study to estimate atmospheric parameters is available at https://atmcorr.gsfc.nasa.gov/ [57,60]. This calculator extracts required parameters from NCEP database using MODTRAN codes and spectral response curve of Landsat 8, Landsat 7, or Landsat 6. Coll et al. [61] have validated this web-based tool against ground measurements for Landsat 7 and reported that the atmospheric correction from this tool is comparable with correction from local radiosonde profiles. The tool was also used to validate a newly proposed pixel-by-pixel atmospheric correction method called SBAC for Landsat 7 in Reference [62]. The online atmospheric correction tool requires the user to input some mandatory data including location (longitude and latitude), date, and time for which the atmospheric parameters are to calculate. Optionally some surface conditions can be input, but if left empty, are assumed from the atmospheric database. The calculated parameters ($\tau_{i}$, $L_{\mathrm{up}}$, and $L_{\mathrm{down}}$) are sent to the user via email.

### 3.2. Single-Channel Algorithm by Jiménez-Muñoz et al. (2014) with Coefficients

In the Single-Channel algorithm, only one thermal band is used to retrieve LST. For Landsat 8, either band 10 or band 11 can be used. The SC algorithm used in this study was developed by Jiménez-Muñoz and Sobrino [36] and was validated for AVHRR channels 4 and 5, ATSR2 channels 1 and 2, and Landsat TM band 6 data. Later this method was adapted for Landsat 8 by Jiménez-Muñoz et al. [37]. According to them, the SC algorithm for Landsat 8 can be expressed as

(4) $$\mathrm{LST}=\gamma \left[\frac{1}{\varepsilon }\left(\psi_{1}L_{\lambda ,\;\mathrm{ToA}}+\psi_{2}\right)+\psi_{3}\right]+\delta ,$$

where ε is the land surface emissivity (LSE); $\psi_{1}$, $\psi_{2}$, and $\psi_{3}$ are atmospheric functions (AFs). The symbols δ and γ represent two parameters that can be estimated from linear approximation of Planck functions as [21]:

(5) $$\gamma ={\left\{\frac{c_{2}\;\cdot \;L_{\lambda ,\mathrm{ToA}}}{T_{\mathrm{ToA}}^{2}}\left[\frac{\lambda^{4}\;\cdot \;L_{\lambda ,\mathrm{ToA}}}{c_{1}}+\frac{1}{\lambda }\right]\right\}}^{-1}.$$

The above equation can be rewritten for simplification as

(6) $$\gamma =\frac{c_{1}\;\cdot \;\lambda \;\cdot \;T_{\mathrm{ToA}}^{2}}{c_{2}\;\cdot \;L_{\lambda ,\mathrm{ToA}}\left(\lambda^{5}\;\cdot \;L_{\lambda ,\mathrm{ToA}}+c_{1}\right)},$$

where $c_{1}$, $c_{2}$, and λ holds same meaning as described in Section 3.1. The parameter δ in Equation (4) can be calculated according to Reference [21] as

(7) $$\delta =-\gamma \;\cdot \;L_{\lambda ,\mathrm{ToA}}+T_{\mathrm{ToA}}.$$

The AFs ($\psi_{1}$, $\psi_{2}$, and $\psi_{3}$) can be approximated with a second-order polynomial fit against atmospheric column water vapor content (*w*). If atmospheric function Ψ is considered as a function of water vapor content *W* as Ψ=CW, the matrix notation to determine the values of AFs can be expressed according to Reference [42] as

(8) $$\left[\begin{array}{c} \psi_{1}\\ \psi_{2}\\ \psi_{3} \end{array}\right]=\left[\begin{array}{ccc} c_{11} & c_{12} & c_{13}\\ c_{21} & c_{22} & c_{23}\\ c_{31} & c_{32} & c_{33} \end{array}\right]\left[\begin{array}{c} w^{2}\\ w\\ 1 \end{array}\right],$$

where $c_{ij}$ are coefficients that were determined by Jiménez-Muñoz et al. [37] for Landsat 8 TIR band 10 using the GAPRI_4838 database as

(9) $$C=\left[\begin{array}{ccc} 0.04019 & 0.02916 & 1.01523\\ -0.38333 & -1.50294 & -0.20324\\ 0.00918 & 1.36072 & -0.27514 \end{array}\right].$$

Combining Equations (8) and (9), we can write

(10) $$\begin{array}{cc} \psi_{1} & =0.04019w^{2}+0.02916w+1.01523, \end{array}$$

(11) $$\begin{array}{cc} \psi_{2} & =-0.38333w^{2}-1.50294w+0.20324, \end{array}$$

(12) $$\begin{array}{cc} \psi_{3} & =0.00918w^{2}+1.36072w-0.27514. \end{array}$$

Using Equations (10)–(12), for a given water vapor content (*w*) the atmospheric functions (AFs) for Single-Channel algorithm can be calculated and then input in the algorithm expressed in Equation (4) to retrieve LST from Landsat 8. Considering different amounts of water vapor content (*w*), the values for $\psi_{1}$, $\psi_{2}$, and $\psi_{3}$ can be calculated as shown in Table 2.

It should be mentioned here that the Equation (4) gives LST products in K (Kelvin) unit when Equation 32 is used to calculate the ToA brightness temperature ($T_{\mathrm{ToA}}$). In order to get the LST in ${}^{{}^{\circ}}C$ (degree Celsius) unit, the $T_{\mathrm{ToA}}$ needs to be calculated in ${}^{{}^{\circ}}C$ unit using Equation (33), which involves the subtraction of 273.15. This $T_{\mathrm{ToA}}$ should then be used to compute the γ and δ parameters. Another way to get the LST products in ${}^{{}^{\circ}}C$ unit is to subtract 273.15 directly in Equation (4).

### 3.3. Split-Window Algorithm by Jiménez-Muñoz et al. (2014) with Coefficients

The Split-Window Algorithm by Jiménez-Muñoz et al. (2014) is based on the mathematical structure by Sobrino et al. [38]; it was later modified by Sobrino and Raissouni [63]. The same mathematical expression was used by Jiménez-Muñoz and Sobrino [58] to retrieve LST from different low-resolution thermal data. Then, it was adapted for Landsat 8 by Jiménez-Muñoz et al. [37]. According to this Split-Window algorithm, LST can be estimated with the following expression:

(13) $$\begin{array}{cc} \mathrm{LST}=T_{i}+c_{0}+c_{1}\left(T_{i}-T_{j}\right) & +c_{2}{\left(T_{i}-T_{j}\right)}^{2}+\left(c_{3}+c_{4}w\right)\left(1-\varepsilon_{m}\right)+\left(c_{5}+c_{6}w\right)\Delta \varepsilon , \end{array}$$

where $T_{i}$ and $T_{j}$ are ToA brightness temperature for band *i* and band *j*. For Landsat 8, these two bands are band 10 and band 11, respectively; $\varepsilon_{m}$ is the mean land surface emissivity of two bands and is calculated with $\frac{1}{2}\left({\mathrm{LSE}}_{10}+{\mathrm{LSE}}_{11}\right)$; Δε is the difference in LSE of two bands, calculated as $\Delta \mathrm{LSE}={\mathrm{LSE}}_{10}-{\mathrm{LSE}}_{11}$; $c_{0},c_{1},\ldots ,c_{6}$ are algorithm coefficients derived from atmospheric simulation codes; *w* is the total atmospheric water vapor content in $g\;cm^{-2}$ unit. For Landsat 8 TIR bands 10 and 11, Equation (13) can be rewritten as

(14) $$\begin{array}{cc} \mathrm{LST}=T_{\mathrm{ToA}\;10}+c_{0}+c_{1}\left(T_{\mathrm{ToA}\;10}-T_{\mathrm{ToA}\;11}\right) & +c_{2}{\left(T_{\mathrm{ToA}\;10}-T_{\mathrm{ToA}\;11}\right)}^{2}\\ \; & +\left(c_{3}+c_{4}w\right)\left(1-{\mathrm{LSE}}_{\mathrm{mean}}\right)+\left(c_{5}+c_{6}w\right)\Delta \mathrm{LSE}. \end{array}$$

The coefficients ($c_{0},c_{1},\ldots ,c_{6}$) for this algorithm, specifically for Landsat 8 data, are given by Jiménez-Muñoz et al. [37]. They have used the GAPRI database to estimate the coefficients using MODTRAN simulation codes. Further details regarding GAPRI database can be found in [37]; determination procedure for algorithm coefficients from different atmospheric databases can be found in [42,58].

The seven coefficient values for Jiménez-Muñoz et al.’s Split-Window algorithm are presented in Table 3. The atmospheric water vapor content (*w*) is not included during the determination of coefficients; it has to be put in the mathematical Expression (14) of the algorithm directly.

### 3.4. Split-Window Algorithm by Du et al. (2015) with Coefficients

The Split-Window Algorithm for Landsat 8 data proposed by Du et al. (2015) [40] is based on the generalized Split-Window algorithm by Wan [39], which was developed for MODerate Resolution Imaging Spectrometer (MODIS) data. The Du et al. algorithm was developed specifically for Landsat 8 data; they named this method as practical Split-Window Algorithm. The mathematical expression of the algorithm is [40]:

(15) $$\mathrm{LST}=b_{0}+\left(b_{1}+b_{2}\frac{1-\varepsilon_{m}}{\varepsilon_{m}}+b_{3}\frac{\Delta \varepsilon }{\varepsilon^{2}}\right)\frac{T_{i}+T_{j}}{2}+\left(b_{4}+b_{5}\frac{1-\varepsilon_{m}}{\varepsilon_{m}}+b_{6}\frac{\Delta \varepsilon }{\varepsilon^{2}}\right)\frac{T_{i}-T_{j}}{2}+b_{7}{\left(T_{i}-T_{j}\right)}^{2}.$$

where $b_{0},b_{1},\ldots b_{7}$ are algorithm coefficients derived from atmospheric profile dataset using simulation codes. All other parameters in Equation (15) are same as in Equation (13), except the *w* in Equation (13), which is absent in Equation (15).

Equation (15) for Landsat 8 data, with more specific expressions, can be rewritten as

(16) $$\begin{array}{cc} \mathrm{LST}=b_{0} & +\left(b_{1}+b_{2}\frac{1-{\mathrm{LSE}}_{\mathrm{mean}}}{{\mathrm{LSE}}_{\mathrm{mean}}}+b_{3}\frac{\Delta \mathrm{LSE}}{{\left(\mathrm{LSE}\right)}^{2}}\right)\frac{T_{\mathrm{ToA}\;10}+T_{\mathrm{ToA}\;11}}{2}\\ \; & +\left(b_{4}+b_{5}\frac{1-{\mathrm{LSE}}_{\mathrm{mean}}}{{\mathrm{LSE}}_{\mathrm{mean}}}+b_{6}\frac{\Delta \mathrm{LSE}}{{\left(\mathrm{LSE}\right)}^{2}}\right)\frac{T_{\mathrm{ToA}\;10}-T_{\mathrm{ToA}\;11}}{2}+b_{7}{\left(T_{\mathrm{ToA}\;10}-T_{\mathrm{ToA}\;11}\right)}^{2}. \end{array}$$

Other than the algorithm coefficients, parameters in this equation are same as in Equation (14). The coefficients ($b_{0},b_{1},\ldots b_{7}$) for this algorithm were estimated by Du et al. [40] under different atmospheric and surface conditions, through numerical simulation, and using atmospheric profiles. As atmospheric profile they have used the TIGR database; and to perform the numerical simulation they used the MODTRAN 5.2 atmospheric transmittance/radiance codes. They used water vapor content (*w*) divided into five sub-ranges to calculate the coefficients. Under different atmospheric conditions, users can use the coefficients for their needed sub-ranges. Further details regarding the procedure of algorithm coefficients’ estimation can be found in [40]. The coefficients for Du et al. algorithm covering different *w* sub-ranges are presented in Table 4.

Using the two Split-Window LST retrieval algorithms in Equations (14) and (16), along with their respective coefficients, LST can be determined once the column water vapor (*w*) of the study area, ToA brightness temperature ($T_{\mathrm{ToA}}$) for both thermal bands, and LSE information is available. The $T_{\mathrm{ToA}}$ is usually estimated from the thermal bands of remote sensing data while the LSE is determined using optical bands as described in the following sections.

### 3.5. Normalized Difference Vegetation Index and the Proportion of Vegetation Cover

In scientific studies of land resources, vegetation index is often used to express the amount of living plant coverage on lands. The term to mathematically express this indication is called Normalized Difference Vegetation Index, or NDVI for short. It is calculated from the visible red and near-infrared (NIR) light reflected by vegetation and can be expressed as

(17) $${\mathrm{NDVI}}_{\mathrm{DN}}=\frac{\mathrm{NIR}-\mathrm{Red}}{\mathrm{NIR}+\mathrm{Red}},$$

where the subscript “DN” stands for “Digital Number”; it implies that the calculation was made using level-1 DN values stored in remote sensing images. As remote sensing images are subject to cloud covers, atmospheric scattering effects, viewing angle problem, etc., the raw RS data need to be converted into surface reflectance values correcting those effects. In practice, the level-1 DN values are first converted into top-of-atmosphere (ToA) spectral reflectance ($\rho_{\lambda ,\mathrm{ToA}}$). It is a unitless quantity and can be calculated from OLI data of Landsat 8 as [48]:

(18) $$\rho_{\lambda ,\mathrm{ToA}(\theta )}=M_{\rho }\times Q_{\mathrm{cal}}+A_{\rho },$$

where $M_{\rho }$ is the reflectance multiplicative scaling factor for the given band, available in the image metadata file as REFLECTANCE_MULT_BAND_*n*, with *n* being 1 through 9; $A_{\rho }$ is the reflectance additive scaling factor for the given band, available in the same file as REFLECTANCE_ADD_BAND_*n*, with *n* being 1 through 9; $Q_{\mathrm{cal}}$ is the level-1 pixel value stored as DN values in the image for both OLI and TIR bands; the θ on left hand side means that this reflectance ($\rho_{\lambda ,\mathrm{ToA}(\theta )}$) does not involve the correction for sun elevation angle.

Making the correction for sun elevation angle, we can exclude the θ from reflectance notation and express the reflectance as [48]:

(19) $$\rho_{\lambda ,\mathrm{ToA}}=\frac{\rho_{\lambda ,\mathrm{ToA}(\theta )}}{\sin\theta },$$

where $\rho_{\lambda ,\mathrm{ToA}(\theta )}$ is the ToA spectral reflectance without sun elevation angle correction as calculated in Equation (18); θ is solar elevation angle, which is the local sun elevation angle at the time of satellite overpass available in the image metadata file of Landsat 8 as SUN_ELEVATION. This value needs to be checked for each Landsat 8 scene. The sun elevation angle can be related with solar zenith angle $\left(\theta_{z}\right)$ using cosine function as

(20) $$\rho_{\lambda ,\mathrm{ToA}}=\frac{\rho_{\lambda ,\mathrm{ToA}(\theta )}}{\cos(\theta_{z})},$$

where $\theta_{z}$ is the solar zenith angle with $\theta_{z}=90^{{}^{\circ}}-\theta$. Combining Equations (18) and (19), we can calculate ToA spectral reflectance as

(21) $$\rho_{\lambda ,\mathrm{ToA}}=\frac{M_{\rho }\times Q_{\mathrm{cal}}+A_{\rho }}{\sin\theta }.$$

To determine NDVI, we need the reflectance values for red and near-infrared bands; this translates to band 4 and band 5 of Landsat 8 image, respectively. The red band in Landsat 8 covers 0.636 to 0.673 μm and NIR band 0.851 to 0.879 μm in the electromagnetic spectrum [48]. For band 4 and band 5, $M_{\rho }$ value is $2.0000\times 10^{-5}$, and $A_{\rho }$ value is −0.100000 [48], as given in the image metadata file.

Once the reflectance is corrected for the sun elevation angle, it needs another correction—the atmospheric correction because the ToA spectral reflectance includes atmospheric scattering effects and therefore does not represent the true reflectance of land surfaces. In order to get the reflectance from the surface ($\rho_{\lambda ,\mathrm{LS}}$), we need to correct atmospheric effects including the cloud cover and atmospheric gases. This correction can be made using some atmospheric correction modules available in various image processing software. Then, surface reflectance can be determined using Equation (21).

To simplify the notation of surface reflectance, in the following we used ρ in place of $\rho_{\lambda ,\mathrm{LS}}$. According to this notation, the NDVI of land surface (${\mathrm{NDVI}}_{\mathrm{LS}}$) can be expressed as

(22) $${\mathrm{NDVI}}_{\mathrm{LS}}=\frac{\rho_{\mathrm{NIR}}-\rho_{\mathrm{red}}}{\rho_{\mathrm{NIR}}+\rho_{\mathrm{red}}}.$$

For Landsat 8 image data, Equation (22) takes the form:

(23) $${\mathrm{NDVI}}_{\mathrm{LS}}=\frac{\rho_{\mathrm{band}\;5}-\rho_{\mathrm{band}\;4}}{\rho_{\mathrm{band}\;5}+\rho_{\mathrm{band}\;4}}.$$

Using ${\mathrm{NDVI}}_{\mathrm{LS}}$ values as in Equation (23), the proportion of vegetation cover can be calculated as [64]:

(24) $$P_{v}={\left(\frac{{\mathrm{NDVI}}_{\mathrm{LS}}-{\mathrm{NDVI}}_{\min}}{{\mathrm{NDVI}}_{\max}-{\mathrm{NDVI}}_{\min}}\right)}^{\;2}\;,$$

where NDVI${}_{\max}=0.5$ indicates the presence of vegetation on lands, and NDVI${}_{\min}=0.2$ represents only bare soil on land surfaces. NDVI value less than 0 indicates the water, and NDVI value greater than 0.5 indicates full vegetation [65]. When NDVI value ranges between 0.2 and 0.5, the surface is considered as a mixture of soil and vegetation, requiring the calculation of $P_{v}$ using Equation (24). Using the $P_{v}$, the emissivity of the mixed land surface is then estimated as described in the following Section.

### 3.6. Land Surface Emissivity Determination

Quantitatively, emissivity is the ratio of the thermal radiation from a surface to the radiation from an ideal black surface at the same temperature as given by the Stefan–Boltzmann law [1]. To retrieve LST from ToA brightness temperature (see Section 3.7), we must consider emissivity from land surfaces. The term Land Surface Emissivity (LSE) is used to indicate emissivity from land surfaces that are composed of different types of materials (soils, vegetation, water etc.). One way to get LSE is through normalized difference vegetation index since NDVI represents the greenness of land surfaces, giving an idea of types of materials comprising the land surfaces. Thus, different values of NDVI represent different materials of land surfaces. If the NDVI value is less than 0.2, then it represents only bare soil. In this case, the emissivity can be calculated from reflectivity values in red region of the image. If the NDVI value is greater than 0.5, then the land surface is composed of vegetation only. If that is the case in a real study, a constant value of emissivity, typically 0.99, can be used [21]. But in situations when NDVI lies between 0.2 and 0.5, LSE for a given band *i* can be related with NDVI and proportion of vegetation ($P_{v}$) as [41]:

(25) $${\mathrm{LSE}}_{i}=\left\{\begin{array}{cc} a_{i}\rho_{\mathrm{red}}+b_{i}\; & \mathrm{NDVI}<0.2\\ \varepsilon_{v,i}P_{v}+\varepsilon_{s,i}\left(1-P_{v}\right)+C_{i}\; & 0.2\leq \mathrm{NDVI}\leq 0.5\\ \varepsilon_{v,i}+C_{i}\; & \mathrm{NDVI}>0.5, \end{array}\right.$$

where $\varepsilon_{v,i}$ is the emissivity of fully vegetated surfaces and $\varepsilon_{s,i}$ is emissivity of barren soil, in the band *i*; $P_{v}$ is the proportion of vegetation as calculated in Equation (24); $a_{i}$, $b_{i}$ are the coefficients that can be estimated from laboratory spectra of soils using statistical fits, assuming that the emissivity and the reflectivities in red band have a linear relationship [66]. The symbol $C_{i}$ in the above equation denotes the roughness of land surfaces [65]. For plain and homogeneous land surfaces, this $C_{i}$ can be neglected [21], and considered $C_{i}=0$ [65]. For rough and heterogeneous surfaces, i.e., soil-vegetation mixed pixels, $C_{i}$ denotes the increment in emissivity resulted from the cavity effect and multiple scattering in the mixed pixels [67].

Taking emissivity values of soil and vegetation into account, and assuming that NDVI values of earth surfaces range from around 0.2 to 0.5, the emissivity of land surfaces (LSE) can be calculated according to the NDVI-threshold method as [41,66]:

(26) $$\mathrm{LSE}=\varepsilon_{v}P_{v}+\varepsilon_{s}\left(1-P_{v}\right)+C.$$

The *C* in the above equation is the same as in Equation (25), which can be found also as dε in literature [21].

According to Equation (26), to calculate LSE for Landsat 8 thermal bands, we need the $\varepsilon_{s}$ and $\varepsilon_{v}$ values estimated for both TIR bands. Yu et al. [41] estimated these values using the MODIS UCSB (University of California, Santa Barbara, CA, USA) emissivity library (https://icess.eri.ucsb.edu/modis/EMIS/html/em.html), as presented in Table 5.

In Equation (26), an approximation of *C* is given by [41,68]:

(27) $$C=\left(1-\varepsilon_{s}\right)\left(1-P_{v}\right)\;F\varepsilon_{v},$$

where *F* is a shape factor [69]. Sobrino et al. [21] considered this shape factor (*F*) under different geometrical distributions having a mean value of 0.55.

Taking Equations (26) and (27) into account, the LSE can be calculated as

(28) $$\mathrm{LSE}=m\;P_{v}+n,$$

with

$$m=\varepsilon_{v}-\varepsilon_{s}-\left(1-\varepsilon_{s}\right)\;F\varepsilon_{v}\;\mathrm{and}\;n=\varepsilon_{s}+\left(1-\varepsilon_{s}\right)\;F\varepsilon_{v}.$$

Based on the emissivity values in Table 5 and the mathematical expression in Equation (28), the LSE for both TIR bands of Landsat 8 data can be calculated. To do so, we first need to calculate the *m* and *n* values for these bands. For TIR band 10, we get

$$\begin{array}{cc} m_{\mathrm{TIR}\;10} & =0.9863-0.9668-(1-0.9668)\times 0.55\times 0.9863\approx 0.0015,\\ n_{\mathrm{TIR}\;10} & =0.9668+(1-0.9668)\times 0.55\times 0.9863\approx 0.9848. \end{array}$$

Thus, using Equation (28), we get the LSE for TIR band 10 as

(29) $${\mathrm{LSE}}_{\mathrm{TIR}\;10}=0.0015\;P_{v}+0.9848.$$

Similarly, for TIR band 11, we get

$$\begin{array}{cc} m_{\mathrm{TIR}\;11} & =0.9896-0.9747-(1-0.9747)\times 0.55\times 0.9896\approx 0.0011,\\ n_{\mathrm{TIR}\;11} & =0.9747+(1-0.9747)\times 0.55\times 0.9896\approx 0.9885. \end{array}$$

Therefore, LSE for TIR band 11 can be expressed as

(30) $$\begin{array}{cc} {\mathrm{LSE}}_{\mathrm{TIR}\;11} & =0.0011\;P_{v}+0.9885. \end{array}$$

Using the proportion of vegetation cover as described in Section 3.5 and expressed in Equation (24), LSE for TIR band 10 and band 11 can be calculated using Equations (29) and (30), respectively.

### 3.7. Top-of-Atmosphere (ToA) Brightness Temperature Determination

Determination of ToA brightness temperature ($T_{\mathrm{ToA}}$) can be described as a two-step process. The first step includes the conversion of level-1 DN values of Landsat 8 thermal infrared data to at-satellite (or at-sensor, or ToA) spectral radiance values. This is the spectral radiance in wavelength of a surface, which is expressed with $L_{\lambda ,\mathrm{ToA}}$ and has a unit of watt per meter squared per steradian per micro meter ($W\;m^{-2}{\mathrm{sr}}^{-1}{\mu m}^{-1}$). The formula to convert level-1 DN values in RS images to spectral radiance is [48]:

(31) $$L_{\lambda ,\mathrm{ToA}}=M_{L}\times Q_{\mathrm{cal}}+A_{L},$$

where $M_{L}$ is the radiance multiplicative scaling factor for the given band, available in the image metadata file as RADIANCE_MULT_BAND_*n*, with *n* being 1 through 11; $A_{L}$ is the radiance additive scaling factor for the given band, available in the same file as RADIANCE_ADD_BAND_*n*, with *n* being 1 through 11; $Q_{\mathrm{cal}}$ is the level-1 pixel value stored as DN values in the image, available for both OLI and TIRS bands. The $M_{L}$ value for both TIR bands (band 10 and band 11) is $3.3420\times 10^{-4}$; and $A_{L}$ value for both of these bands is 0.10000. Spectral radiance can be obtained for both OLI and TIR bands but in order to retrieve LST from Landsat 8 thermal bands, radiance from only thermal bands is necessary. For both TIR bands, $L_{\mathrm{ToA}\;10}$ and $L_{\mathrm{ToA}\;11}$ can be estimated following Equation (31).

The second step to determine $T_{\mathrm{ToA}}$ involves the use of $L_{\lambda ,\mathrm{ToA}}$ image data from Equation (31). Then, $T_{\mathrm{ToA}}$ in K (Kelvin) unit can be calculated by inverting Planck’s radiation equation as [48]:

(32) $$T_{\mathrm{ToA}}=\frac{K_{2}}{\ln\left(\frac{K_{1}}{L_{\lambda ,\mathrm{ToA}}}+1\right)},$$

where $K_{1}$ and $K_{2}$ are the thermal conversion constants for the given band, available in the image metadata file as K1_CONSTANT_BAND_*n* and K2_CONSTANT_BAND_*n*, respectively, with *n* being 10 or 11; $L_{\lambda ,\mathrm{ToA}}$ is the ToA spectral radiance calculated for band 10 or band 11 with Equation (31). The $K_{1}$ and $K_{2}$ are numerical constant values for TIR bands of Landsat 8; for band 10 the values are 774.8853 and 1321.0789, respectively; for band 11 they are 480.8883 and 1201.1442, respectively.

The ToA brightness temperature in ${}^{{}^{\circ}}C$ (degree Celsius) unit can be estimated by subtracting 273.15 in Equation (32) as

(33) $$T_{\mathrm{ToA}}=\frac{K_{2}}{\ln\left(\frac{K_{1}}{L_{\lambda ,\mathrm{ToA}}}+1\right)}-273.15.$$

According to Equation (33), the $T_{\mathrm{ToA}\;10}$ and $T_{\mathrm{ToA}\;11}$ for both TIR bands of Landsat 8 can be estimated in ${}^{{}^{\circ}}C$ unit.

### 3.8. Preparation and Processing of Data for LST Retrieval from Landsat 8

In order to perform atmospheric corrections of Landsat 8 data, as well as for other processing of raster images, different computer tools were used in this study. First, appropriate Landsat 8 images were downloaded from the website of USGS earth explorer. Vector shapefiles were downloaded from the GADM website (http://gadm.org/data.html). Spatial subset of the raster image was created using the shapefile for our study area in QGIS [70] software. Atmospheric corrections including the removal of cloud and haze from the level-1 Landsat 8 data were done using the ATCOR module in PCI Geomatica 2016 software. From the atmospherically corrected subset image data, NDVI was determined using raster calculator of QGIS. Other calculations, for example, proportion of vegetation cover, LSE, LST, etc. were also estimated with the same tool. The LST maps were exported using the print composer function of QGIS. Statistical analyses and all types of plots and histograms were created using the R program [71] with substantial help from the raster [72], rgdal [73], ggplot2 [74], and caret [75] libraries.

## 4. Results and Discussion

The LST products retrieved from all four algorithms were validated against reference LST (${\mathrm{LST}}_{\mathrm{ref}}$) and cross-validated against MODIS daily LST (${\mathrm{LST}}_{\mathrm{MOD}}$). The RMSE (root-mean-squared-error) was used to measure the accuracy of LST retrieved with four methods comparing them against reference LST and MODIS LST. The reference LSTs for all Landsat 8 images were estimated using the ATCOR module available in PCI Geomatica 2016 software. The MODIS daily LST was retrieved using the AρρEEARS online application from the Terra MODIS images. The online atmospheric correction calculator (https://atmcorr.gsfc.nasa.gov/) was used for the estimation of atmospheric parameters, including the water vapor content. For several locations in the study area, a total of 48 calculations of *w* for each Landsat 8 scene were made using the online tool and the *w* range was found to be 0.6 to 3.0 g ${\mathrm{cm}}^{-2}$ considering all five Landsat 8 images. Therefore, we have used several values of *w* for LST retrieval.

The LST result retrieved with the RTE-based direct method is denoted as ${\mathrm{LST}}_{\mathrm{RTE}}$, LST with Single-Channel algorithm as ${\mathrm{LST}}_{\mathrm{SC}}$, and LSTs with two Split-Window algorithms as ${\mathrm{LST}}_{\mathrm{Jim}}$ (for Jiménez-Muñoz et al.’s method), and ${\mathrm{LST}}_{\mathrm{Du}}$ (for Du et al.’s method). In the following, the LST results from all four algorithms for the Landsat 8 image of 21 February 2018 (Section 4.1, Section 4.2, Section 4.3 and Section 4.4) are presented along with an intercomparison study among them (Section 4.5). In addition, variation in LST results with varied amount of *w* is discussed in Section 4.6. Intercomparison results from the cross-validation of Landsat 8 LSTs against MODIS daily LSTs are presented in Section 4.7.

### 4.1. Results from RTE Method using TIR Band 10

**Figure 4:**
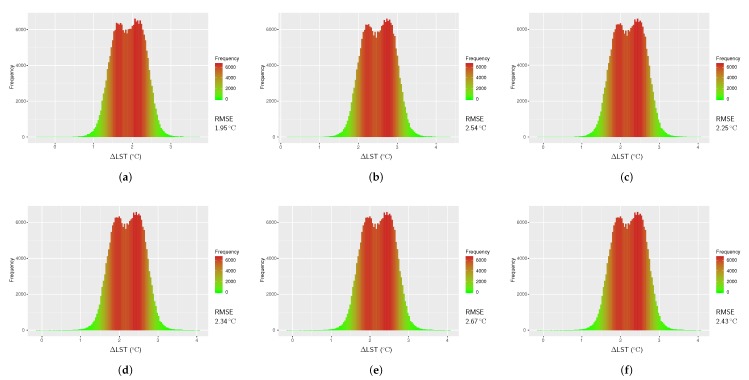
Histograms of temperature difference between the reference LST (${\mathrm{LST}}_{\mathrm{ref}}$) and LST from Radiative Transfer Equation (RTE)-based algorithm (${\mathrm{LST}}_{\mathrm{RTE}}$) for Landsat 8 image of 21 February 2018; (**a**) τ=0.76, $L_{\mathrm{up}}=1.97$, $L_{\mathrm{down}}=3.23$, (**b**) τ=0.78, $L_{\mathrm{up}}=1.85$, $L_{\mathrm{down}}=3.04$, (**c**) τ=0.77, $L_{\mathrm{up}}=1.91$, $L_{\mathrm{down}}=3.14$, (**d**) τ=0.77, $L_{\mathrm{up}}=1.92$, $L_{\mathrm{down}}=3.16$, (**e**) τ=0.76, $L_{\mathrm{up}}=1.94$, $L_{\mathrm{down}}=3.19$, and (**f**) τ=0.77, $L_{\mathrm{up}}=1.93$, $L_{\mathrm{down}}=3.17$ ($L_{\mathrm{up}}$ and $L_{\mathrm{down}}$ are in W m^−2^sr^−1^μm^−1^ unit).

The atmospheric parameters required to retrieve LST in direct method using RTE (3) were obtained using the online atmospheric correction calculator for six specific locations of the study area as presented in Table 6. The values in several observations were found to be close to each other. In order to compute the RMSE, we first calculated ΔLST comparing the RTE-retrieved LST and the reference LST. The validation results are represented with histograms in Figure 4 including the RMSE values. The smallest RMSE for LST${}_{\mathrm{RTE}}$ was found to be 1.95 ${}^{{}^{\circ}}C$, with the largest being 2.67 ${}^{{}^{\circ}}C$.

### 4.2. Results from Single-Channel Algorithm using TIR Band 10

**Figure 5:**
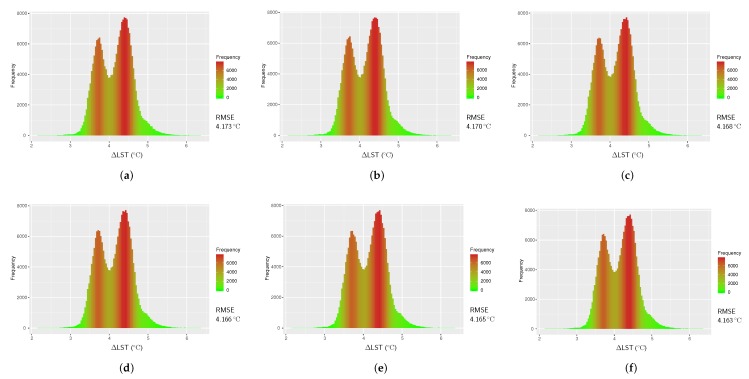
Histograms of temperature difference between the reference LST (${\mathrm{LST}}_{\mathrm{ref}}$) and LST from Single-Channel algorithm (${\mathrm{LST}}_{\mathrm{SC}}$) for Landsat 8 image of 21 February 2018; (**a**) w=0.5 g cm^−2^, (**b**) w=1.0 g cm^−2^, (**c**) w=1.5 g cm^−2^, (**d**) w=2.0 g cm^−2^, (**e**) w=2.5 g cm^−2^, and (**f**) w=3.0 g cm^−2^.

The LST retrieved with Single-Channel algorithm from band 10 of Landsat 8 uses Equation (4) and atmospheric functions calculated for different values of water vapor content as given in Table 2. For our study area, we retrieved LSTs for *w* range of 0.5 to 3.0 g cm^−2^ with an interval of 0.5 g cm^−2^. This gives LST calculations for six values of *w*. All LST products obtained in this method were then validated against reference LST as shown in Figure 5 with their RMSEs. The smallest RMSE was found to be 4.16 ${}^{{}^{\circ}}C$ for w=3.0 g cm^−2^, whereas the largest RMSE was 4.17 ${}^{{}^{\circ}}C$ for w=0.5 g cm^−2^.

### 4.3. Results from Jiménez-Muñoz et al.’s Split-Window Algorithm

**Figure 6:**
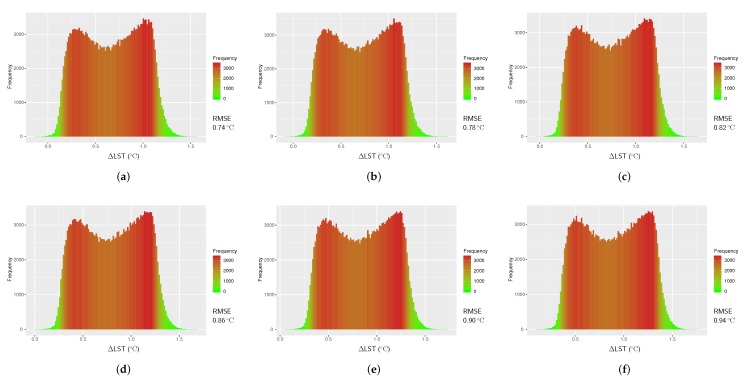
Histograms of temperature difference between the reference LST (${\mathrm{LST}}_{\mathrm{ref}}$) and LST from Jiménez-Muñoz et al.’s Split-Window algorithm (${\mathrm{LST}}_{\mathrm{Jim}}$) for Landsat 8 image of 21 February 2018; (**a**) w=0.5 g cm^−2^, (**b**) w=1.0 g cm^−2^, (**c**) w=1.5 g cm^−2^, (**d**) w=2.0 g cm^−2^, (**e**) w=2.5 g cm^−2^, and (**f**) w=3.0 g cm^−2^.

The coefficient values in Jiménez-Muñoz et al.’s (2014) algorithm (see Table 3) do not include the water vapor content (*w*); instead, the algorithm in Equation (14) takes the *w* as a direct input from the user. Like in Single-Channel algorithm, we used six values of *w* to retrieve LSTs in this method (${\mathrm{LST}}_{\mathrm{Jim}}$). To compute RMSEs, all observations of ${\mathrm{LST}}_{\mathrm{Jim}}$ were validated against reference LST and presented as histograms in Figure 6. The smallest RMSE for ${\mathrm{LST}}_{\mathrm{Jim}}$ was found to be 0.74 ${}^{{}^{\circ}}C$ when w=0.5 g cm^−2^, with the largest RMSE being 0.94 ${}^{{}^{\circ}}C$ when w=3.0 g cm^−2^.

### 4.4. Results from Du et al.’s Split-Window Algorithm

**Figure 7:**
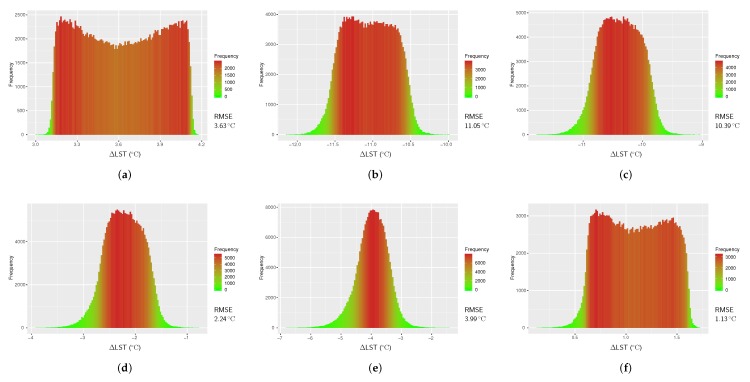
Histograms of temperature difference between the reference LST (${\mathrm{LST}}_{\mathrm{ref}}$) and LST from Du et al.’s Split-Window algorithm (${\mathrm{LST}}_{\mathrm{Du}}$) for Landsat 8 image of 21 February 2018; (**a**) w= 0.0 to 2.5 g cm^−2^, (**b**) w= 2.0 to 3.5 g cm^−2^, (**c**) w= 3.0 to 4.5 g cm^−2^, (**d**) w= 4.0 to 5.5 g cm^−2^, (**e**) w= 5.0 to 6.3 g cm^−2^, and (**f**) w= 0.0 to 6.3 g cm^−2^.

As it is mentioned previously, the coefficient values in Du et al.’s (2015) algorithm were estimated using water vapor contents (*w*) of various sub-ranges (see Table 4). Therefore, LSTs were estimated with this algorithm (${\mathrm{LST}}_{\mathrm{Du}}$) using algorithm coefficients given for *w* sub-ranges of 0.0 to 2.5 g cm^−2^, 2.0 to 3.5 g cm^−2^, 3.0 to 4.5 g cm^−2^, 4.0 to 5.5 g cm^−2^, 5.0 to 6.3 g cm^−2^, and 0.0 to 6.3 g cm^−2^. All six LSTs were then validated against reference LST and RMSEs were computed (Figure 7). The smallest RMSE ( 1.13 ${}^{{}^{\circ}}C$) was found when LST was estimated using *w* in 0.0 to 6.3 g cm^−2^ while in *w* range of 2.0 to 3.5 g cm^−2^ the LST was found with abnormally the largest RMSE ( 11.05 ${}^{{}^{\circ}}C$). This error could be due to the wrong use of *w* amount (see Section 4.6).

### 4.5. Intercomparison of Four Algorithms to Retrieve LST from Landsat 8 Against Reference LSTs

The LST results as estimated from the Landsat 8 image of 21 February 2018 with all four algorithms (see Section 4.1, Section 4.2, Section 4.3 and Section 4.4) were compared among them validating the results against reference LST by means of RMSE computation and $R^{2}$ (coefficient of determination). In addition, algorithm-retrieved LSTs were also observed against reference LST by means of mean bias error (${\mathrm{Bias}}_{\mathrm{ref}}$).

Except for ${\mathrm{LST}}_{\mathrm{RTE}}$, a sub-range of *w* was considered for the average LST values for three other methods (${\mathrm{LST}}_{\mathrm{SC}}$, ${\mathrm{LST}}_{\mathrm{Jim}}$, and ${\mathrm{LST}}_{\mathrm{Du}}$). Considering the water vapor content of 1.68 to 2.43 g cm^−2^ for the 21 February 2018 Landsat 8 image, as retrieved from the NCEP database with MODTRAN codes, a *w* range of 1.5 to 3.0 g cm^−2^ was considered as the possible mean of *w* for ${\mathrm{LST}}_{\mathrm{SC}}$ and ${\mathrm{LST}}_{\mathrm{Jim}}$ methods. For the ${\mathrm{LST}}_{\mathrm{Du}}$ method, the algorithm coefficients available in *w* range of 0.0 to 6.3 g cm^−2^, that is, the entire range of *w* was considered.

The minimum and maximum LST, along with the statistical mean and standard deviation of LST retrieved with four methods, and the reference LST are presented in Table 7. The RMSE and $R^{2}$, as well as the mean bias (Bias${}_{\mathrm{ref}}$) of algorithm-retrieved LSTs computed against reference LST, are also presented.

As seen in Table 7, the mean RMSE was found 2.20 ${}^{{}^{\circ}}C$ for ${\mathrm{LST}}_{\mathrm{RTE}}$; 4.17 ${}^{{}^{\circ}}C$ for ${\mathrm{LST}}_{\mathrm{SC}}$; 0.88 ${}^{{}^{\circ}}C$ for ${\mathrm{LST}}_{\mathrm{Jim}}$; and 1.13 ${}^{{}^{\circ}}C$ for ${\mathrm{LST}}_{\mathrm{Du}}$. The average correlation of coefficient ($R^{2}$) for ${\mathrm{LST}}_{\mathrm{Jim}}$ and ${\mathrm{LST}}_{\mathrm{Du}}$ is 0.86; it is 0.77 for ${\mathrm{LST}}_{\mathrm{RTE}}$, and 0.76 for ${\mathrm{LST}}_{\mathrm{SC}}$ method. This indication implies that all four algorithms perform efficiently compared to reference LST. The mean bias against reference LST (bias${}_{\mathrm{ref}}$) is the smallest in ${\mathrm{LST}}_{\mathrm{Jim}}$ ( 0.82 ${}^{{}^{\circ}}C$) and largest in ${\mathrm{LST}}_{\mathrm{SC}}$ ( 4.14 ${}^{{}^{\circ}}C$), with ${\mathrm{LST}}_{\mathrm{Du}}$ ( 1.08 ${}^{{}^{\circ}}C$) and ${\mathrm{LST}}_{\mathrm{RTE}}$ ( 2.30 ${}^{{}^{\circ}}C$) being in between. Considering all these observations, the best performing LST algorithm in our study is ${\mathrm{LST}}_{\mathrm{Jim}}$, with the other three methods staying in close agreement.

**Figure 8:**
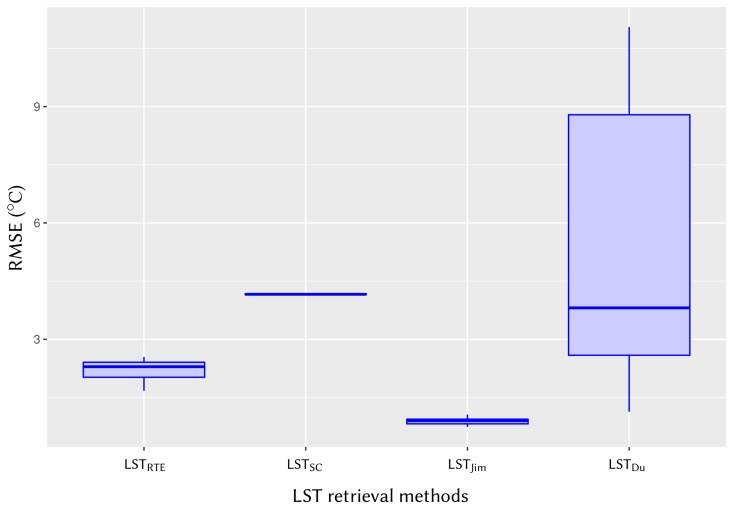
Box plots showing RMSEs of four LST retrieval algorithms as estimated on the Landsat 8 image of 21 February 2018.

The box plots in Figure 8 represent LST results of four algorithms in terms of RMSE, giving a visual aid for intercomparison. As seen in this figure, variation in RMSE is lowest for ${\mathrm{LST}}_{\mathrm{Jim}}$, with ${\mathrm{LST}}_{\mathrm{RTE}}$ and ${\mathrm{LST}}_{\mathrm{SC}}$ being next to it and ${\mathrm{LST}}_{\mathrm{Du}}$ having the highest variation. The rather different RMSE variation in ${\mathrm{LST}}_{\mathrm{Du}}$ is probably due to the wrong use of *w*, which is described in Section 4.6.

**Figure 9:**
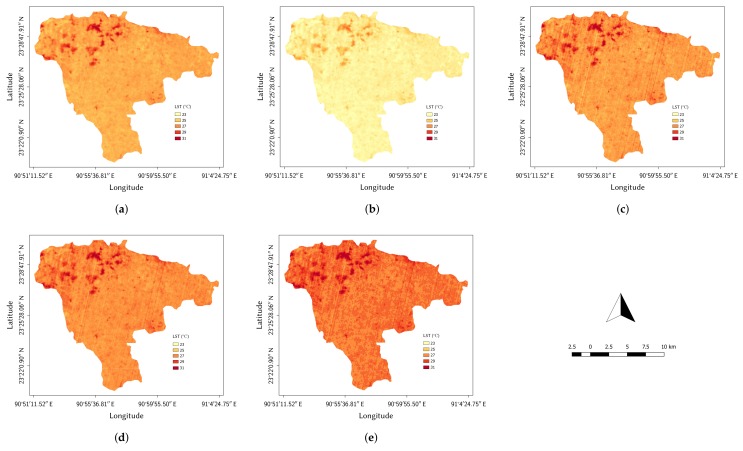
LST maps of the agricultural study area (Landsat 8 image of 21 February 2018): (**a**) RTE-based method using TIR band 10 (τ=0.76, $L_{\mathrm{up}}=1.94$ W m^−2^sr^−1^μm^−1^, $L_{\mathrm{down}}=3.19$ W m^−2^sr^−1^μm^−1^), (**b**) Single-Channel algorithm using TIR band 10 (w=2.0 g cm^−2^), (**c**) Du et al.’s [40] Split-Window algorithm (w= 0.0 to 6.3 g cm^−2^), (**d**) Jiménez-Muñoz et al.’s [37] Split-Window algorithm (w= 1.0 g cm^−2^), and (**e**) using the ATCOR module (the reference LST).

The LST maps created using all four methods and with ATCOR module (LST${}_{\mathrm{ref}}$) are shown in Figure 9. We presented the maps with LST results that were found very close to the reference LST, considering the possible amount of *w* present in our study area. This makes the LST maps for ${\mathrm{LST}}_{\mathrm{SC}}$ and ${\mathrm{LST}}_{\mathrm{Jim}}$ computed with w=2.0 g cm^−2^, and ${\mathrm{LST}}_{\mathrm{Du}}$ computed with *w* range of 0.0 to 6.3 g cm^−2^.

**Figure 10:**
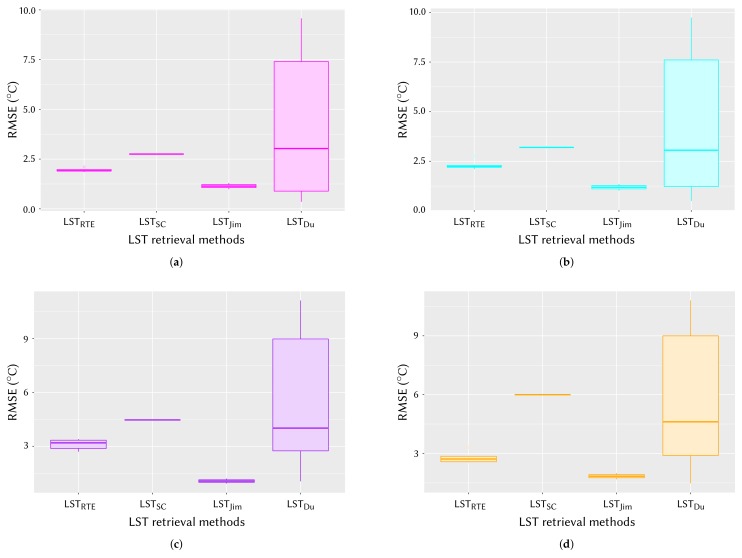
Box plots showing RMSEs of four LST retrieval algorithms as obtained from four Landsat 8 images: (**a**) 4 January 2018 image, (**b**) 20 January 2018 image, (**c**) 9 March 2018 image, and (**d**) 25 March 2018 image.

Similar intercomparison study was performed on four additional Landsat 8 images. The box plots representing the intercomparison results of four LST algorithms on Landsat 8 images of different dates are shown in Figure 10. As seen in this figure, the LST results of different algorithms obtained for several Landsat 8 images agree with the results obtained for Landsat 8 image of 21 February 2018 (see Figure 8).

Considering all five Landsat 8 images used in this intercomparison study, the average RMSE is found to be 2.47 ${}^{{}^{\circ}}C$ for the ${\mathrm{LST}}_{\mathrm{RTE}}$ method; 4.11 ${}^{{}^{\circ}}C$ for ${\mathrm{LST}}_{\mathrm{SC}}$; 1.19 ${}^{{}^{\circ}}C$ for ${\mathrm{LST}}_{\mathrm{Jim}}$; and 1.50 ${}^{{}^{\circ}}C$ for the ${\mathrm{LST}}_{\mathrm{Du}}$ algorithm (not shown in Table). It can be mentioned here that the *w* values retrieved from the NCEP database were different for Landsat 8 images of different dates (see Table 8). The corresponding *w* ranges were taken into consideration for the calculation of LSTs with different methods.

### 4.6. Variation in LST Results due to Wrong Amount of Water Vapor Contents

The Single-Channel algorithm and the two Split-Window algorithms used to retrieve LST from Landsat 8 are dependent on atmospheric water vapor content (*w*). Therefore, it is necessary to study their performance in different amounts of *w*. To perform this study, we retrieved LSTs for the Single-Channel algorithm and Jiménez-Muñoz et al.’s Split-Window algorithm for *w* amount up to 4.5 g cm^−2^, that is, three more observations than it is seen in Table 7. For Du et al.’s Split-Window algorithm, we used the LSTs retrieved for all *w* sub-ranges.

**Figure 11:**
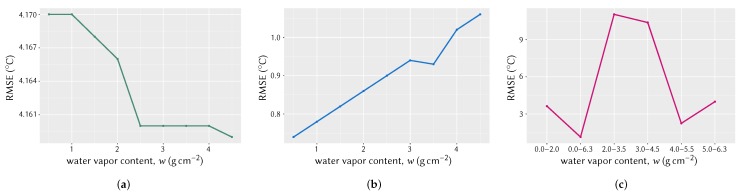
Variation of RMSEs with water vapor content: (**a**) using Single-Channel algorithm for Landsat 8 TIR band 10, (**b**) Split-Window algorithm and coefficients according to Jiménez-Muñoz et al., and (**c**) Split-Window algorithm and coefficients according to Du et al.

The variation in RMSEs of LST results for three algorithms was plotted against varied amount of *w* as shown in Figure 11. Since the algorithms for ${\mathrm{LST}}_{\mathrm{SC}}$ and ${\mathrm{LST}}_{\mathrm{Jim}}$ require direct input of *w*, the two plots (Figure 11a,b) share *w* values in same intervals. On the other hand, RMSE variation for the ${\mathrm{LST}}_{\mathrm{Du}}$ algorithm is shown with several sub-ranges of *w* (Figure 11c).

As seen in Figure 11a, for ${\mathrm{LST}}_{\mathrm{SC}}$ the RMSE decreases with increasing *w*, whereas in Figure 11b, RMSE for ${\mathrm{LST}}_{\mathrm{Jim}}$ increases with increasing *w*. The decrease of RMSE for ${\mathrm{LST}}_{\mathrm{SC}}$ in lower *w* is quite abrupt compared to its change in higher *w* (Figure 11a). For the ${\mathrm{LST}}_{\mathrm{Jim}}$, the increase in RMSE with increasing *w* is almost constant (Figure 11b). On the other hand, for ${\mathrm{LST}}_{\mathrm{Du}}$ (Figure 11c), LST error is very high when w= 0.0 to 4.5 g cm^−2^; it is lowest when *w* lies in 4.0 to 6.3 g cm^−2^; it is considerably better when w= 0.0 to 6.3 g cm^−2^, that is, algorithm coefficients for the entire *w* range. It is understandable from these plots that the use of wrong *w* value may result in unacceptable LST estimation, especially for the ${\mathrm{LST}}_{\mathrm{Du}}$ algorithm. Therefore, it is very important to estimate the *w* of the study area with great precision.

### 4.7. Cross-Validation and Intercomparison of Landsat 8 LSTs Against MODIS Daily LSTs

**Figure 12:**
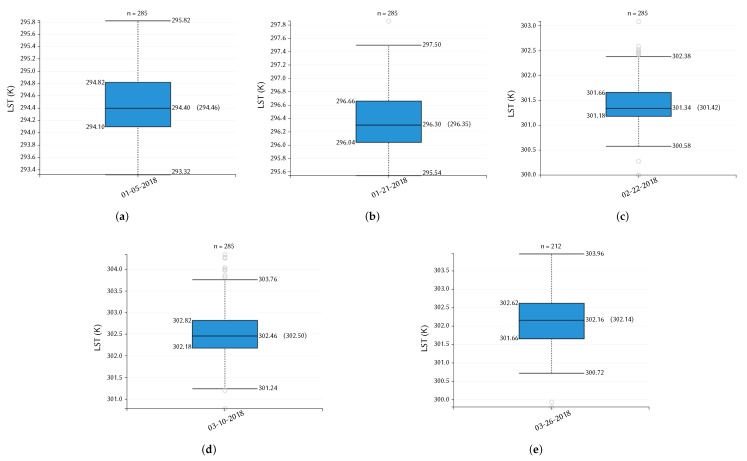
Box plots representing daily LSTs retrieved from the Terra MODIS images of different dates: (**a**) 5 January 2018 image, (**b**) 21 January 2018 image, (**c**) 22 February 2018 image, (**d**) 10 March 2018 image, and (**e**) 26 March 2018 image. The parameter *n* indicates the total number of values/pixels; the boxes show the interquartile range (IQR) along with median, mean (in parentheses) values; the whiskers represent the lowest and highest datum within 1.5 IQR of the lower- and upper-quartile, respectively (all LST values are in K unit).

As mentioned previously, MODIS daily LSTs for the study area were extracted using the AρρEEARS online tool. We considered the nearest available LSTs compared with Landsat 8 images of different dates used in this study. Since there are missing values for daily LSTs due to the effects of clouds and other atmospheric conditions, we retrieved MODIS LSTs for the dates of: (a) 5 January 2018, (b) 21 January 2018, (c) 22 February 2018, (d) 10 March 2018, and (e) 26 March 2018. The statistical values including the median and mean MODIS LSTs retrieved for these dates are presented in Figure 12 using box plots.

For cross-validation of Landsat 8 LSTs that were retrieved with four algorithms, as well as with the ATCOR module, the MODIS daily LSTs were converted into ${}^{{}^{\circ}}C$ unit from K unit. Then, LST mean bias was computed for each Landsat 8 LST retrieval method against MODIS mean LST. The cross-validation and intercomparison results between Landsat 8 and MODIS daily LSTs are presented in Table 8. The Landsat 8 mean LST is denoted with ${\mathrm{LST}}_{L8}$, MODIS mean LST with ${\mathrm{LST}}_{\mathrm{MOD}}$, and the mean LST bias between them is denoted with Bias${}_{\mathrm{MOD}-L8}$.

As seen in Table 8, the mean bias (Bias${}_{\mathrm{MOD}-L8}$) between ${\mathrm{LST}}_{L8}$ and ${\mathrm{LST}}_{\mathrm{MOD}}$ is always lower for the ${\mathrm{LST}}_{\mathrm{Jim}}$ algorithm among four Landsat 8 LST algorithms. The ${\mathrm{LST}}_{\mathrm{Du}}$ algorithm performs very close to the ${\mathrm{LST}}_{\mathrm{Jim}}$ method. The ${\mathrm{LST}}_{\mathrm{RTE}}$ shows higher LST bias while the ${\mathrm{LST}}_{\mathrm{SC}}$ has the highest bias among these four methods. It suggests that the best performing LST retrieval method for Landsat 8 is the ${\mathrm{LST}}_{\mathrm{Jim}}$ Split-Window algorithm, with ${\mathrm{LST}}_{\mathrm{Du}}$ method being the next, and ${\mathrm{LST}}_{\mathrm{RTE}}$ having better performance than the ${\mathrm{LST}}_{\mathrm{SC}}$ method. These cross-validation and intercomparison results agree with the intercomparison results obtained for different Landsat 8 LST methods when validated against ${\mathrm{LST}}_{\mathrm{ref}}$ (see Section 4.5). It also reveals that the reference LST estimated from ATCOR module (${\mathrm{LST}}_{\mathrm{ref}}$) performs efficiently showing a very small mean bias (from −0.58 to −0.29 ${}^{{}^{\circ}}C$) for most images and the lowest mean bias for the first three Landsat 8 images (Table 8) used in this study (the only exception is the Landsat 8 image of 25 March 2018 with its corresponding MODIS daily LST where the mean bias is considerably higher).

## 5. Conclusions

Landsat 8 data are great sources of high resolution remote sensing images with two thermal bands that can be efficiently used to retrieve LST. An intercomparison study among the existing LST algorithms for Landsat 8 was performed against ATCOR-derived reference LSTs and AρρEEARS-derived Terra MODIS daily LSTs. According to the observation of this study, the Single-Channel algorithm can be used in LST retrieval for Landsat 8 images, but use of a good Split-Window algorithm has the potential of ensuring greater accuracy. The challenges with a good, practical, and feasible Split-Window algorithm development can be the precise estimation of coefficient values and determination of atmospheric water vapor content of the study area.

Taking all Landsat 8 images used in this study under consideration against reference LST, the RTE-based method (${\mathrm{LST}}_{\mathrm{RTE}}$) gives LST results better than the Single-Channel method (${\mathrm{LST}}_{\mathrm{SC}}$) with an average $\mathrm{RMSE}=2.47\;{}^{{}^{\circ}}C$; but it (${\mathrm{LST}}_{\mathrm{RTE}}$) performs worse compared to the Split-Window algorithms. Since the RTE-based direct algorithm depends heavily on various atmospheric parameters, precision calculation of those parameters can be the determinant of LST accuracy. The use of NCEP database and MODTRAN codes through the online atmospheric correction calculator seems promising for this method.

The LST results with Single-Channel algorithm provide larger RMSE (average $\mathrm{RMSE}=4.11\;{}^{{}^{\circ}}C$) than the RTE-based method. In contrast, Jiménez-Muñoz et al.’s Split-Window Algorithm (${\mathrm{LST}}_{\mathrm{Jim}}$) shows the best performance (average $\mathrm{RMSE}=1.19\;{}^{{}^{\circ}}C$) among other methods. These two LST algorithms (${\mathrm{LST}}_{\mathrm{SC}}$ and ${\mathrm{LST}}_{\mathrm{Jim}}$) can be chosen when: (a) actual water vapor content is precisely measured, and (b) other atmospheric parameters are not available as they are not necessary in these two methods. Especially for the Single-Channel algorithm, it is not advisable to use this method for images that have more than one thermal band.

The Du et al.’s Split-Window Algorithm (${\mathrm{LST}}_{\mathrm{Du}}$) with its coefficients available for *w* range 0.0 to 6.3 g cm^−2^ can be applied for an area where the actual amount of water vapor content cannot be determined or very uncertain for precision determination. The reasons behind this recommendation are: (a) this algorithm gives good LST results compared to other methods with $\mathrm{RMSE}=1.50\;{}^{{}^{\circ}}C$ as found in this study, and (b) other three LST algorithms require either the direct input of water vapor content (${\mathrm{LST}}_{\mathrm{SC}}$ and ${\mathrm{LST}}_{\mathrm{Jim}}$ methods) or several atmospheric parameters (${\mathrm{LST}}_{\mathrm{RTE}}$ method). On the other hand, the potential problem with this method is that the *w* range of 0.0 to 6.3 g cm^−2^ is too much of generalization and could give unsatisfactory results in areas with different atmospheric conditions than observed in this study.

The cross-validation and intercomparison results of Landsat 8 LSTs with different algorithms against MODIS daily LSTs were found to agree with the intercomparison results against reference LSTs. The mean bias (Bias${}_{\mathrm{MOD}-L8}$) here for the ${\mathrm{LST}}_{\mathrm{Jim}}$ algorithm was found always lower compared to other Landsat 8 LST algorithms. The ATCOR-derived Landsat 8 LST was found with even lower Bias${}_{\mathrm{MOD}-L8}$ for the first three images used in this study revealing that the ATCOR-derived LSTs can be used as references for the indirect verification of Landsat 8 LST algorithms.

The in situ LSTs were not available in this study; therefore, ground validation of Landsat 8 LST algorithms was not performed. Monitoring of in situ LST data with precision radiosounding instruments or radiometers correcting for the effects of emissivity and synchronizing with the actual time of satellite overpass could be taken into consideration to perform the ground validation of LST algorithms.

Although the NCEP atmospheric profile database was found providing with good estimation of atmospheric parameters, other databases (e.g., TIGR, GAPRI, CLAR, etc.) can be used to study their relative performances. Land surface emissivity from MODIS or ASTER remote sensing images can be compared against NDVI-based emissivity in the retrieval of precision LST. Cross-validation of LST from Landsat 8 with MODIS daily LST retrieved using the AρρEEARS online tool was found promising in the intercomparison study of different Landsat 8 LST algorithms. Other sources of remote sensing data can be used in the cross-validation study to further enhance the verification of Landsat 8 LST products.

## Figures and Tables

**Table 1:** Landsat 8 and Terra MODerate Resolution Imaging Spectroradiometer (MODIS) image data used in this study.

Landsat 8 Images				MODIS Images		
Image Date	Scene Identifier	Path	Row	Image Date	Identifier	Data Type
4 January 2018	LC81370442018004LGN00	137	44	5 January 2018	MOD11A1.006	LST_Day_1km
20 January 2018	LC81370442018020LGN00	137	44	21 January 2018	MOD11A1.006	LST_Day_1km
21 February 2018	LC81370442018052LGN00	137	44	22 February 2018	MOD11A1.006	LST_Day_1km
9 March 2018	LC81370442018068LGN00	137	44	10 March 2018	MOD11A1.006	LST_Day_1km
25 March 2018	LC81370442018084LGN00	137	44	26 March 2018	MOD11A1.006	LST_Day_1km

**Table 2:** Atmospheric functions calculated for different amount of water vapor contents following Equations (10)–(12).

*w* (g cm${}^{-2}$)	$\psi_{1}$	$\psi_{2}$	$\psi_{3}$
0.5	1.039858	−0.6440625	0.407515
1.0	1.08458	−1.68303	1.09476
1.5	1.149398	−2.913663	1.786595
2.0	1.23431	−4.33596	2.48302
2.5	1.339317	−5.949922	3.184035
3.0	1.46442	−7.75555	3.88964
3.5	1.609618	−9.752843	4.599835
4.0	1.77491	−11.9418	5.31462
4.5	1.960298	−14.32242	6.033995

**Table 3:** Split-Window Algorithm coefficients for Jiménez-Muñoz et al.’s (2014) algorithm as obtained from numerical simulation. Source: Reference [37]. RMSE = Root-mean-squared-error.

Coefficient	Value	RMSE
$c_{0}$	−0.268	0.984
$c_{1}$	1.378	
$c_{2}$	0.183	
$c_{3}$	54.30	
$c_{4}$	−2.238	
$c_{5}$	−129.20	
$c_{6}$	16.40	

**Table 4:** Split-Window Algorithm coefficients for Du et al.’s (2015) method as estimated from numerical simulation. Source: Reference [40].

*w* (g cm${}^{-2}$)	$b_{0}$	$b_{1}$	$b_{2}$	$b_{3}$	$b_{4}$	$b_{5}$	$b_{6}$	$b_{7}$	RMSE (K)
[0.0, 2.5]	−2.78009	1.01408	0.15833	−0.34991	4.04487	3.55414	−8.88394	0.09152	0.34
[2.0, 3.5]	11.00824	0.95995	0.17243	−0.28852	7.11492	0.42684	−6.62025	−0.06381	0.60
[3.0, 4.5]	9.62610	0.96202	0.13834	−0.17262	7.87883	5.17910	−13.26611	−0.07603	0.71
[4.0, 5.5]	0.61258	0.99124	0.10051	−0.09664	7.85758	6.86626	−15.00742	−0.01185	0.86
[5.0, 6.3]	−0.34808	0.98123	0.05599	−0.03518	11.96444	9.06710	−14.74085	−0.20471	0.93
[0.0, 6.3]	−0.41165	1.00522	0.14543	−0.27297	4.06655	−6.92512	−18.27461	−0.24468	0.87

**Table 5:** Emissivity values of soil and vegetation for TIR band 10 and band 11. Source: Reference [41].

	Emissivity Values	
TIR Band	Vegetation ($\varepsilon_{v}$)	Soil ($\varepsilon_{s}$)
band 10	0.9863	0.9668
band 11	0.9896	0.9747

**Table 6:** Atmospheric parameters retrieved through online atmospheric correction calculator for the Landsat 8 image of 21 February 2018 ($L_{\mathrm{up}}$ and $L_{\mathrm{down}}$ are in W m^−2^sr^−1^μm^−1^ unit).

Location		Atmospheric Parameters			
Latitude	Longitude	τ	$L_{\mathrm{up}}$	$L_{\mathrm{down}}$	*w* (g cm${}^{-2}$)
23°20′ N	91°00′ E	0.76	1.97	3.23	1.87–2.32
23°20′ N	91°22′ E	0.78	1.85	3.04	1.68–2.32
23°27′ N	90°57′ E	0.77	1.91	3.14	1.87–2.43
23°24′ N	90°55′ E	0.77	1.92	3.16	1.87–2.43
23°28′ N	91°00′ E	0.76	1.94	3.19	1.87–2.32
23°21′ N	90°56′ E	0.77	1.93	3.17	1.87–2.41

**Table 7:** Intercomparison results of LSTs retrieved with four algorithms from Landsat 8 data (21 February 2018) against reference LSTs.

		LST Estimates (${}^{{}^{\circ}}$C)				Comparison Results		
LST Method	*w* (g cm${}^{-2}$)	MIN	MAX	MEAN	SD	RMSE (${}^{{}^{\circ}}$C)	$R^{2}$	Bias ${}_{\mathrm{ref}}$ (${}^{{}^{\circ}}$C)
LST${}_{\mathrm{ref}}$	–	25.00	34.00	27.80	0.8237	–	–	–
LST${}_{\mathrm{RTE}(\mathrm{avg})}$	1.68–2.43	24.00	31.33	25.50	0.7081	2.20	0.77	2.30
LST${}_{\mathrm{SC}}$	0.5	22.38	28.13	23.65	0.5542	4.17	0.76	4.15
	1.0	22.38	28.14	23.65	0.5545	4.17	0.76	4.15
	1.5	22.38	28.14	23.66	0.5548	4.17	0.76	4.15
	2.0	22.38	28.15	23.66	0.5552	4.17	0.76	4.14
	2.5	22.38	28.15	23.66	0.5557	4.16	0.76	4.14
	3.0	22.38	28.16	23.66	0.5562	4.16	0.76	4.14
	3.5	22.38	28.17	23.66	0.5568	4.16	0.76	4.14
	4.0	22.38	28.18	23.66	0.5575	4.16	0.76	4.14
	4.5	22.38	28.19	23.67	0.5583	4.16	0.76	4.14
LST${}_{\mathrm{SC}(\mathrm{avg})}$	1.5–3.0	22.38	28.15	23.66	0.5555	4.17	0.76	4.14
LST${}_{\mathrm{Jim}}$	0.5	24.99	32.83	27.13	0.7106	0.74	0.86	0.67
	1.0	24.94	32.75	27.09	0.7103	0.78	0.86	0.71
	1.5	24.93	32.63	27.04	0.7101	0.82	0.86	0.76
	2.0	24.87	32.70	27.00	0.7098	0.86	0.86	0.80
	2.5	24.83	32.65	26.96	0.7095	0.90	0.86	0.84
	3.0	24.80	32.61	26.92	0.7093	0.94	0.86	0.88
	3.5	24.76	32.57	26.87	0.7090	0.93	0.86	0.93
	4.0	24.73	32.52	26.83	0.7088	1.02	0.86	0.97
	4.5	24.69	32.48	26.79	0.7085	1.06	0.86	1.01
LST${}_{\mathrm{Jim}(\mathrm{avg})}$	1.5–3.0	24.86	32.65	26.98	0.7097	0.88	0.86	0.82
LST${}_{\mathrm{Du}}$	0.0–2.5	21.94	30.24	24.18	0.7514	3.63	0.87	3.62
	2.0–3.5	35.97	45.41	38.85	0.8432	11.05	0.86	−11.05
	3.0–4.5	35.06	45.06	38.18	0.8929	10.39	0.85	−10.38
	4.0–5.5	26.75	37.23	30.01	0.9350	2.24	0.85	−2.21
	5.0–6.3	27.51	39.98	31.76	1.1160	3.99	0.82	−3.96
	0.0–6.3	24.23	33.23	26.72	0.8070	1.13	0.86	1.08

**Table 8:** Cross-validation and intercomparison results of Landsat 8 LSTs retrieved with four algorithms and with the Atmospheric and Topographic CORection (ATCOR) module against Terra MODIS LSTs. All LST values are in ${}^{{}^{\circ}}C$; image acquisition dates are in yy-mm-dd format. Notations for different LST retrieval methods from Landsat 8 data are the same as in previous sections.

Landsat 8 LSTs				Terra MODIS LSTs		
Image Date	LST Methods	MEAN ${\mathrm{LST}}_{L8}$	*w* (g cm${}^{-2}$)	Image Date	MEAN ${\mathrm{LST}}_{\mathrm{MOD}}$	Bias${}_{\mathrm{MOD}-L8}$
2018-01-04	${\mathrm{LST}}_{\mathrm{RTE}}$	19.65	0.5–2.0	2018-01-05	21.31	1.66
	${\mathrm{LST}}_{\mathrm{SC}}$	18.84				2.47
	${\mathrm{LST}}_{\mathrm{Jim}}$	20.56				0.75
	${\mathrm{LST}}_{\mathrm{Du}}$	19.67				1.64
	${\mathrm{LST}}_{\mathrm{ref}}$	21.60				−0.29
2018-01-20	${\mathrm{LST}}_{\mathrm{RTE}}$	21.31	0.5–2.0	2018-01-21	23.20	1.89
	${\mathrm{LST}}_{\mathrm{SC}}$	20.41				2.79
	${\mathrm{LST}}_{\mathrm{Jim}}$	22.54				0.66
	${\mathrm{LST}}_{\mathrm{Du}}$	21.67				1.53
	${\mathrm{LST}}_{\mathrm{ref}}$	23.58				−0.38
2018-02-21	${\mathrm{LST}}_{\mathrm{RTE}}$	25.64	1.5–3.0	2018-02-22	28.27	2.63
	${\mathrm{LST}}_{\mathrm{SC}}$	23.66				4.61
	${\mathrm{LST}}_{\mathrm{Jim}}$	26.98				1.29
	${\mathrm{LST}}_{\mathrm{Du}}$	26.72				1.55
	${\mathrm{LST}}_{\mathrm{ref}}$	27.80				0.47
2018-03-09	${\mathrm{LST}}_{\mathrm{RTE}}$	26.89	1.0–2.5	2018-03-10	29.35	2.46
	${\mathrm{LST}}_{\mathrm{SC}}$	25.51				3.84
	${\mathrm{LST}}_{\mathrm{Jim}}$	28.97				0.38
	${\mathrm{LST}}_{\mathrm{Du}}$	28.94				0.41
	${\mathrm{LST}}_{\mathrm{ref}}$	29.93				−0.58
2018-03-25	${\mathrm{LST}}_{\mathrm{RTE}}$	28.93	3.0–4.5	2018-03-26	28.99	0.06
	${\mathrm{LST}}_{\mathrm{SC}}$	25.70				3.29
	${\mathrm{LST}}_{\mathrm{Jim}}$	29.74				−0.75
	${\mathrm{LST}}_{\mathrm{Du}}$	30.23				−1.24
	${\mathrm{LST}}_{\mathrm{ref}}$	31.66				−2.67
